# Multiplex bioimaging of single-cell spatial profiles for precision cancer diagnostics and therapeutics

**DOI:** 10.1038/s41698-020-0114-1

**Published:** 2020-05-01

**Authors:** Mayar Allam, Shuangyi Cai, Ahmet F. Coskun

**Affiliations:** 1 Wallace H. Coulter Department of Biomedical Engineering, Georgia Institute of Technology and Emory University, Atlanta, GA USA; 2 Interdisciplinary Program in Bioengineering, Georgia Institute of Technology, Atlanta, GA USA

**Keywords:** Molecular imaging, Systems biology, Cancer imaging, Mathematics and computing

## Abstract

Cancers exhibit functional and structural diversity in distinct patients. In this mass, normal and malignant cells create tumor microenvironment that is heterogeneous among patients. A residue from primary tumors leaks into the bloodstream as cell clusters and single cells, providing clues about disease progression and therapeutic response. The complexity of these hierarchical microenvironments needs to be elucidated. Although tumors comprise ample cell types, the standard clinical technique is still the histology that is limited to a single marker. Multiplexed imaging technologies open new directions in pathology. Spatially resolved proteomic, genomic, and metabolic profiles of human cancers are now possible at the single-cell level. This perspective discusses spatial bioimaging methods to decipher the cascade of microenvironments in solid and liquid biopsies. A unique synthesis of top-down and bottom-up analysis methods is presented. Spatial multi-omics profiles can be tailored to precision oncology through artificial intelligence. Data-driven patient profiling enables personalized medicine and beyond.

## Introduction

Tumors arise from abnormal cells that acquire uncontrolled proliferation and extensive differentiation abilities^1^. Tumor development is dynamic and evolutionary. To adapt to environmental changes in local cancerous tissues within a timed progression window, tumors acquire spatial and temporal heterogeneity^2^. As a result of this structural and functional complexity of tumors, cancer therapies exhibit variable responses in distinct patients and cancer types. Conventional chemotherapies are prone to fail due to drug resistance. Tumor origin may be associated with foreign infections, genetic causes, cellular diseases, evolutionary formation, and systemic perturbation of homeostasis^3^. As one of these mechanisms for tumor initiation, a cancer cell can be considered as a cancer stem cell, due to its similar features in self-renewal and differentiation of normal stem cells^4^. In the cancer stem cell model, therapeutic reagents may eliminate cancer cells with limited proliferative potential but remain unsuccessful to target multipotent cancerous cells. Cancer relapses after chemotherapies in a majority portion of patients, because the resistance of cancer stem cell to chemotherapies is a primary reason to relapses^5^. Reprogramming of tumor cells during drug treatments may explain drug resistance that leads to relapses^6^. Rare cells develop unexpected epigenetic programs to acquire secondary mutations for stable resistance. Secondary genetic alterations and proteomic bypass mechanisms contribute to the resistance^7^. Therefore, cancer heterogeneity and therapeutic variability indicate the need for personalized medicine, wherein precision treatments are designed based on an individual’s functional molecular profiles^8^.

Personalized medicine benefits from precise molecular profiles of tumors in the form of solid and liquid tumors. Solid tumors are composed of immobile cells, such as epithelial or mesenchymal cells that accumulate multiple mutations. On the other hand, liquid tumors contain mobile and invasive neoplastic cells with less number of mutations^9^. Tumor genotypes are used for therapies in hematologic and solid tumors. The current medical practice focuses on single lesions, wherein invasive tumor biopsies either from the bone marrow or from affected nodal/soft tissue are targeted. However, the single-site tumor biopsies fail to identify the entire mutational profile due to the limited genomic heterogeneity of an individual’s disease. Solid biopsies also cause biases in disease characterization and lead to erroneous therapeutic decisions due to the difference in sampling locations within biopsies. In the meantime, circulating free DNA (cfDNA) has been widely explored since its identification in 1948^10^. Cancer patients have increased levels of DNA fragments in the blood plasma frequently, which are possibly released from apoptotic or necrotic cells^11^. Therefore, circulating tumor DNA (ctDNA) shows the potential to represent genomic biopsy. Compared with single-lesion tissue biopsies, liquid biopsies exhibit better performance to elucidate acquired resistance. Next-generation sequencing (NGS) has enabled profiling of ctDNA as a small fraction of total cfDNA, opening new doors to use of liquid biopsies for disease diagnostics^12,13^.

However, the lower quantity of ctDNA in cfDNA limits the sensitivity of detection and imaging is not the optimum approach for measuring DNA due to low signal levels. In addition to the histological analysis of solid tumors, imaging circulating tumor cells (CTCs), CTC clusters, and immune cells is an alternative way to analyze the tumor’s molecular compositions. CTCs are considered as real-time “liquid biopsy.” Both single CTCs and CTC clusters present heterogeneous molecular characteristics. Also, CTCs in liquid biopsies give a better representation of dynamic immune profiles, such as PD-L1 expression, than tissue biopsies^14^. In addition to PD-L1, the circulating T cells with different T-cell receptors show the potential to be unique biomarkers for immuno-oncology^14^. The heterogeneity presented by both CTCs and immune profiles, and the limited number of CTCs emphasize the need for effective biomarker-based detection methods, thus developing automated, multiplex imaging methods are promising^15^.

**Fig. 1:**
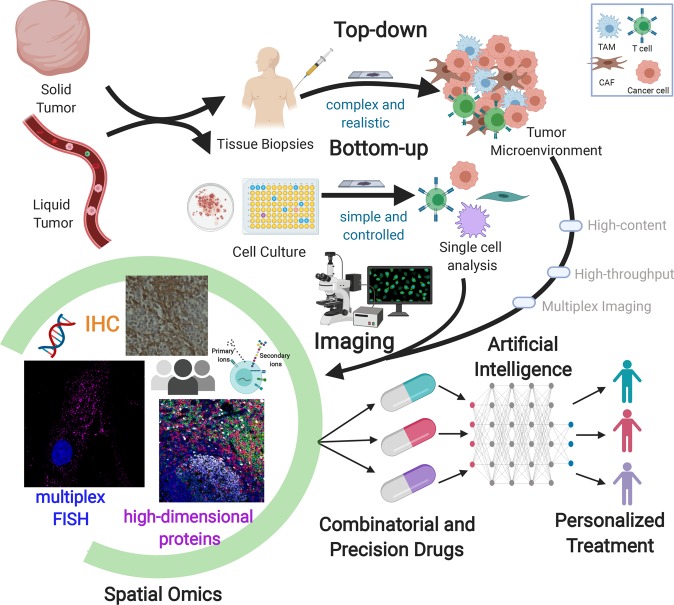
Spatial bioimaging for precision cancer diagnostics at the single-cell and subcellular levels. Two approaches are involved in analyzing solid and liquid tumors. The top-down approach collects human tissue biopsies and studies the tumor heterogeneity from human tissue biopsies, maintaining the architecture of tumor cells. The bottom-up approach generates reproducible data by programming the dynamics in the tumor microenvironment in controllable conditions such as cell cultures. Multiplex imaging techniques that leverage immunohistochemistry (IHC), immunofluorescence (IF), fluorescence in situ hybridization (FISH), multiplexed ion beam imaging (MIBI), and imaging mass cytometry (IMC) can then be applied to both approaches to profile molecular characteristics. From the molecular spatial-omics maps, artificial intelligence categorizes responder and non-responder patient groups to predict the individualized therapeutic treatment for an incoming patient. Created with BioRender.com.

This perspective focuses on the applications of bioimaging technologies to screen patients’ molecular heterogeneity in solid tumors and cellular constituents of liquid biopsies for predictive and personalized medicine applications (Fig. 1). Two mainstream approaches are discussed. The top-down method analyzes the molecular characteristics that are measured in either liquid or solid biopsies, wherein the architecture of the cells is maintained in physiologically relevant conditions, while the bottom-up technique generates highly reproducible data by patterning and modifying the cells to resemble the dynamic features of the native tissue using microwell and microfluidic interfaces^16^. These complementary schemes elucidate the complexity and heterogeneity of tumor microenvironments. Spatial molecular analysis of RNA and protein markers has great potential to dissect cellular constituents and interactions in tumor microenvironments at the tissue level, cell clusters, and subcellular scales. These high-dimensional cellular image-omics profiles may show differences between biopsies from the patients who either respond or resist to therapies. Spatial maps of patient subgroups can be used as training and learning datasets. Artificial intelligence platforms can then predict a new patient’s response to drugs for personalized treatment plans based on the spatial data collected from both top-down and bottom-up approaches. This integrative framework impacts precision medicine and therapeutic design.

## Spatial hierarchical structures for cancer microenvironments

Molecular imaging is a ubiquitous and powerful approach to rapidly monitor the progression of cancers at low cost. Several imaging modalities are used by clinicians to detect, diagnose, and stage human cancers including X-ray, ultrasound, and magnetic resonance imaging (MRI). However, these imaging techniques detect anatomical aberrations at the organ level without specific genetic inference and with limited molecular information^17^. Although positron emission tomography (PET) measures the biochemical activity of cancer cells, it is limited to a few traces that depend on cellular metabolism and that is insufficient to understand the entire molecular profiles of human cancers^18^. Thus, emerging solutions are urgently needed for technological advances in cancer diagnostics for the simultaneous detection of hundreds of biological markers in tumors to decipher a wide range of molecular drivers in human diseases.

Molecular profiles of cancers aid clinicians in designing personalized treatment regimens and potentially open doors to drug discovery research. Complementary imaging methods in histological and pathological routines utilize specific protein-targeting chemistry such as antibodies to detect molecular profiles at the cellular level. Although these microscale imaging efforts have identified vital biomarkers in cancers, they are constrained by the number of markers that can be measured in a biopsy sample. Recent advances utilize multiplex cellular imaging systems that allow visualization of a larger number of markers of up to 50 unique targets. Current research aims to develop automated, multiplexed platforms to detect numerous markers and to feed the information in an algorithm to diagnose patients and to predict their response to therapies.

### Tumor microenvironment

**Fig. 2:**
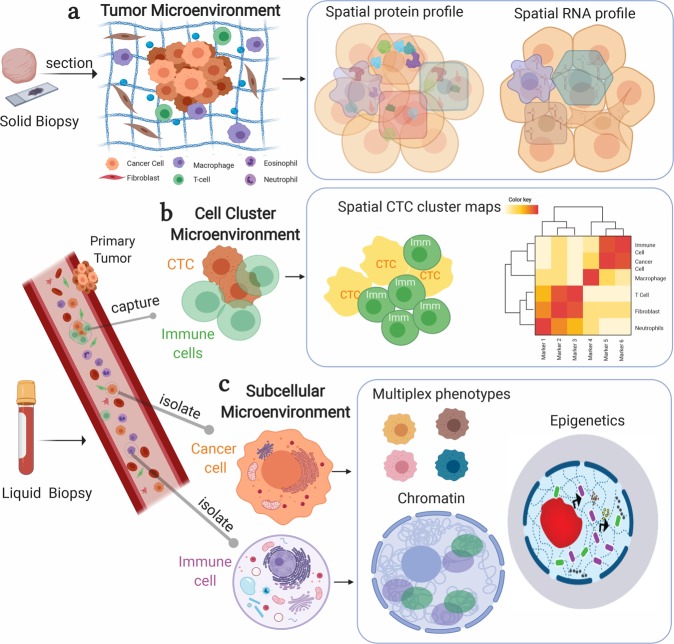
Multiplex bioimaging of hierarchical spatial microenvironments in tumors. **a** Multiplexed imaging techniques can be used in solid biopsies to understand the tissue microenvironment. Spatial protein and RNA profiles can be mapped directly in tissues. **b** Multiplexed imaging can also be used for liquid biopsies to capture and analyze the circulating tumor cells (CTCs) and the immune cells, wherein heterogeneity in cell clusters is studied by heatmaps and clustering. Microfluidic devices are developed for the isolation of CTC clusters, which are then imaged to study the cell cluster microenvironment. **c** Single cancer cells or immune cells are isolated from liquid biopsies. The subcellular microenvironment of single cells can be deciphered using multiplexed analysis. Imaging mass cytometry identified multiplex phenotypes of circulating tumor cells from prostate cancer patients^26^. The subcellular microenvironment can further be studied by subcellular imaging platforms to detect the signatures of chromatin folding and protein factors for distinct epigenetic states. Created with BioRender.com.

Cancer cells are the key constituents of tissue masses in tumors, but they are also surrounded by a complex microenvironment that comprises a heterogeneous mixture of stromal cells, immune cells, cytokines, and extracellular proteins^19^. The tumor microenvironment holds a wealth of information about the biochemical, physical, genetic aberrations in cancers that can be used to design patient-specific therapies. This information often remains unlocked due to the limitation of the commonly used technologies. For example, routine lab assays often fail in maintaining intact specimens of tissue samples or in detecting multiple markers from one sample. Although sequencing and flow cytometry have been informative about tumor microenvironment (TME), spatially resolved profiles of genes and molecular products such as RNAs and proteins are critical to uncovering molecular drivers of cellular interactions in tumors (Fig. 2a)^20^. Thus, a comprehensive study for TMEs necessitates multiplex imaging methods for the regulation of RNA, protein, and metabolite markers in patients.

### Cell cluster microenvironment

Liquid biopsy has tremendous potential in deciphering the genomic and proteomic signatures of the CTCs, which are the cancer cells that escape from the primary tumor into the patient’s bloodstream, and the disseminating tumor cells (DTCs), which are a fraction of CTCs that resides in distant sites^21^. Medical care providers track the disease progression and assess the treatment efficiency in real-time using molecular and cellular profiles of CTCs in liquid biopsies from cancer patients. One of the routine assays that are used by clinicians is CellSearch, the only Food and Drug Administration-approved test to identify, isolate, and enumerate CTCs from a blood sample. However, CellSearch is only utilizing limited markers to identify CTC, resulting in variable results due to the dynamic nature of cancer cells and finally leading to underestimating the density of CTC^22^. Thereby, multiplexed imaging techniques are well-suited to study cellular constituents of liquid biopsies by with higher accuracy and specificity to understand their biological signatures.

CTCs also appear in the form of cell clusters that comprise cancer cells and immune cells. Microfluidic interfaces have been designed to isolate the CTC clusters from blood specimens of cancer patients^23^. These CTC clusters provide information about the tumor’s molecular evolution, their release mechanisms, and location from which they are coming on primary tumors (Fig. 2b). Immune cells in clusters also are related to tissue-derived macrophages and other subtypes of immune phenotypes. These molecular correlative studies between the CTC clusters and primary solid tumors have been performed with RNA sequencing, which provides limited information about their spatial regulation in tumors. Thus, multiplex spatial bioimaging will be powerful to decipher “CTC cluster microenvironments” in metastatic physical progression for designing efficient therapies.

### Subcellular microenvironment

Further, multiplexed imaging techniques can be used to decipher the cancer heterogeneity in multiple levels ranging from cellular clusters and down to the subcellular level. In solid tumors, the “subcellular microenvironment” was measured and quantified to decipher the functional differences of individual cells. For example, a multiplexed imaging mass cytometry (IMC) system detected 32 proteins and phosphorylation sites in formalin-fixed, paraffin-embedded (FFPE) tissue samples from 20 breast cancer patients. Several protein markers were uniquely distributed in their subcellular volumes at different sites of the cancer masses, such as the invasive front, center, and the periphery. The relation between two mRNA transcripts was also explored in subcellular regions that are commonly used as treatment targets (CK19 and HER2) and their corresponding proteins^24,25^. In the tumor microenvironment, a hierarchical regulation from subcellular architecture to tissue composition regulates the cellular response to diseases.

CTCs and immune cells circulate in the bloodstream under the effect of serum and other growth factors. When treated with drugs, these single cells receive messages from the suspension-form micro-niches to respond or resist. Thus, the “subcellular microenvironment” in single cells from liquid biopsies can be profiled for functional analysis. Receptor interactions, signaling networks, and chromatin/protein interactions contribute to these subcellular and molecular microenvironments. Spatially resolved methods achieve a subcellular resolution to assay intracellular molecular distributions. For example, multiplexed IMC detected 16 protein markers associated with phenotypes of CTCs, DTCs, and immune cells from liquid biopsies (blood and bone marrow) from a metastatic prostate cancer patient (Fig. 2c). In this work, CTCs and DTCs were isolated from blood and bone marrow samples, and their genomic profiles were profiled. Patient-specific immune signatures were also observed, which could potentially be used for designing a personalized treatment plan^26^.

Another recent approach was an mRNA detection assay that was used to profile CTCs for analysis of only viable CTCs that contribute to disease progression^27^. Ten markers were combined in an RNA panel for identifying CTCs that are functional in metastatic breast cancer patients. In addition, super-resolution imaging of chromatin state^28^ and spatially resolving protein modifications^29^ in the epigenetic mechanisms further sheds light on subcellular microenvironments (Fig. 2c). Multiplexed and super-resolved bioimaging approaches^30^ are becoming highly critical for functional studies of single cells in solid and liquid biopsies.

## Spatial-omics technologies for precision oncology

Multiplex imaging techniques meet TME studies with their measurement capabilities in native tissues with a large target panel, as summarized in Table 1. These methods detect multiparameter maps of cellular profiles in both fresh-frozen and FFPE biopsies. Two mainstream approaches have been employed for multiplex bioimaging of proteins, which are important markers for functional analysis of cells in TMEs. To perform multiplexed measurements, conventional fluorescence imaging platforms have been combined with repeated labeling of the biopsy samples using chemical bleaching/antibody stripping of labels or DNA barcoded antibodies. CycIF was developed as a cyclic method that used regular antibody and dye conjugates, where a mix of bleaching cocktail (4.5% H_2_O_2_ and 24 mM NaOH in phosphate-buffered saline) was used to remove the signal from the prior cycle, re-labeling of another set of antibodies, followed by imaging and another signal bleaching^31,32^. This process of imaging, bleaching, and re-labeling was then repeated for multiplexed detection. CO-Detection by indEXing (CODEX) was then designed as another sequential labeling method that utilized and rendered dye-labeled DNA sequences that were conjugated to a library of antibodies. CODEX rendering process was repeated up to 15 cycles to anneal and strip DNA barcodes for multiplexing proteins in spleen samples^33^. A recent antibody–DNA barcoding technology, Immuno-SABER (signal amplification by exchange reaction), was combined with a unique signal amplification method for multiplex imaging of lowly expressed markers in tissues^30^. Fluorescence-based multiplexing methods are available for individual laboratories using automated microfluidic and imaging instruments.

**Table 1:** Spatial-omics technologies. Single-cell technologies for spatially resolved measurements in cells and tissues. Multiplex protein imaging is largely targeted technologies based on mass spectrometry and fluorescence cyclic imaging. RNA imaging is performed either at the full genome-scale or targeted panel for a subset of genomic markers. Metabolic imaging was achieved by mass spectrometry imaging at different resolution levels.

Spatial omics	Target	Read-out	Modality	Resolution	Coverage	Refs.
CODEX	Protein	Cyclic imaging	Fluorescence	Optical ( × 20)	Targeted	^33^
MIBI	Protein	Mass Spectrometry	SIMS	260 nm	Targeted	^20,36^
CycIF	Protein	Cyclic imaging	Fluorescence	Optical ( × 20 to × 60)	Targeted	^31,32^
IMC	Protein	Mass Spectrometry	Laser ablation	1 µm	Targeted	^24,25^
MxIF	Protein	Cyclic imaging	Fluorescence	Optical ( × 20)	Targeted	^129^
Immuno-SABER	Protein	Cyclic imaging	Fluorescence	Super Resolution	Targeted	^30^
GeoMx DSP	Protein	Sequencing	Fluorescence	Single cell	Full/targeted	^67^
GeoMx DSP	RNA	Sequencing	Fluorescence	Single cell	Full/targeted	^130^
Spatial transcriptomics	RNA	Sequencing	Fluorescence	100 µm	Full	^37^
SLIDE SEQ	RNA	Sequencing	Fluorescence	10 µm	Full	^40^
HDST	RNA	Sequencing	Fluorescence	2 µm	Full	^41^
DNA Microscopy	RNA	Sequencing	Fluorescence	10 µm	Full	^43^
FISSEQ	RNA	Sequencing	Fluorescence	Nucleotide	Full	^42^
STARMAP	RNA	Cyclic imaging	Fluorescence	Single cell	Targeted	^53^
SEQUANTIAL FISH	RNA	Cyclic imaging	Fluorescence	Optical ( × 60)	Targeted	^44,45^
SEQUENTIAL FISH+	RNA	Cyclic imaging	Fluorescence	Super Resolution	Targeted	^46^
MERFISH	RNA	Cyclic imaging	Fluorescence	Optical ( × 60)	Targeted	^48,49^
osmFISH	RNA	Cyclic imaging	Fluorescence	Optical ( × 100)	Targeted	^131^
SABER-FISH	RNA	Cyclic imaging	Fluorescence	Optical ( × 100)	Targeted	^54^
MALDI	Metabolite	Mass Spectrometry	MALDI	1.4 µm	Discovery	^59^
t-MALDI-2	Metabolite	Mass Spectrometry	t-MALDI-2	0.6 µm pixel	Discovery	^60^
OrbiSIMS	Metabolite	Mass Spectrometry	SIMS	0.3 µm	Discovery	^82^
TOF-SIMS	Metabolite	Mass Spectrometry	SIMS	100 nm	Discovery	^132^
NanoSIMS	Metabolite	Mass Spectrometry	SIMS	50 nm	7-Channel	^61^

*Read-out*: Cyclic imaging is the repeated labeling of the same cells using either automated fluidics or manual protocols. Mass spectrometry and next-generation sequencing allow direct multiplex detection.

*Modality*: The contrast reagent that is detected by an instrumental imager such as fluorescence imaging, secondary ion beam spectrometry (SIMS), Laser ablation, and matrix-assisted laser desorption/ionization (MALDI).

*Resolution*: “Optical (20×)” refers to low-magnification and lower numerical aperture imaging on a fluorescence microscope, while “Optical (60×)” implies significantly higher magnification and higher numerical aperture on the similar microscopes. “Super Resolution” is short for super-resolution microscopy that achieves the highest resolution in the order of sub-100-nm. “Nucleotide” is not an optical resolution but the read-out resolution that is obtained by labeling chemistry. “Single cell” refers to decent cellular detection with limited subcellular details.

*Coverage*: “Full” is the entire genome mapping of a cell without the need for target information, on the other hand, “targeted” requires priori information about panel design to select a subset of the entire genome. “Discovery” also refers to the full molecular mapping of isotope-mass channel without the need for a prior target identity. “7-Channel” denotes technically available channels but not the fundamental limitation of the technology.

Another direction for multiplexing proteins is by imaging of mass-tag labeled antibodies. Isotope labels have created high-dimensional profiles of cellular suspensions from biopsies to decipher cellular regulations by a mass cytometry technique (Cytometry by time-of-flight (TOF)) at the single-cell level^34^. This platform has recently been extended to the spatial mapping of proteins by IMC^24,35^. In this method, laser ablation was used to raster scan the tissue sample, releasing vapors from specimens to be analyzed by a TOF detector for identifying mass-to-charge ratios (elemental types) and abundances of isotopes. As a complementary effort, the second approach is multiplexed ion beam imaging by the TOF (MIBI-TOF). In this technique, an ion beam source was used to raster scan the tissues to generate secondary ions that are detected by a TOF analyzer. MIBI-TOF profiled 36 proteins in triple-negative breast cancer (TNBC) FFPE samples from 41 patients. In this work, a structured view of immune cell populations and checkpoints expression was observed and overall survival was linked to immune profiles in TNBCs^20,36^.

Multiplex visualization of RNA transcripts has been performed at two distinct scales, using either genome-wide or targeted (subset of genome) screens. The first approach has developed sample preparation and barcoding techniques for encoding spatial positions onto the DNA identifier sequence, followed by NGS of positional barcoded transcriptional profiles. In this direction, spatial transcriptomics (ST)^37,38^ has profiled genome-wide transcriptional changes in tissues at sub-100 µm resolution. In the ST platform, a barcoded substrate was prepared and the tissue sample was digested directly on this interface. The resultant cDNA sequences were analyzed by a sequencer. ST was applied to prostate cancers from a high-throughput sample size of 6750 tissue regions, revealing the spatial gradient of stromal cells to tumor regions^39^. The integration of bead chemistry with sequencing has allowed higher resolution mapping of spatially resolved RNA maps. Slide-seq utilized a relatively simplified protocol with 10-µm resolution in the mouse brain and other organs^40^. Another implementation, high-definition ST (HDST), created spatially resolved maps of histological sections at 2 µm resolution^41^. Different enzymatic chemistry was also used to map transcriptome using cDNA conversion based on fluorescent in situ RNA sequencing (FISSEQ) technology^42^. Recent work introduced a reconstruction framework, termed as DNA microscopy^43^, to perform spatially resolved measurements in cells. DNA microscopy allows RNA profiling at the single-cell level based on the relative positioning of biomolecules that are barcoded by unique molecular identifiers. ST and NGS-based transcriptional profiling methods approach subcellular resolution with genome-scale mapping, providing a convenient tool for cancers.

The latter approach has directly profiled a subset of the transcriptome by imaging of hybridized oligonucleotides on transcripts. Multiplexed error-robust fluorescent in situ hybridization (MERFISH) and sequential FISH utilized single-molecule barcoding of individual RNA molecules that were imaged, aligned, and de-barcoded to perform spatially resolved transcriptomics up to 10,000 gene targets^44–49^. These methods have provided transcriptional profiles in cultures and brain slices to reveal the spatial organization of cells. SeqFISH+ deserves special attention as the spatial resolution has been reported at the sub-diffraction-limit, highest resolving power in RNA-profiling approaches^46^. Signal amplification strategies have enabled spatially resolved RNA measurements in breast cancer tissues and larger structures such as zebrafish embryos and thicker brain slices^50–52^. Recently, another ligation chemistry was developed for STARmap technology to reconstruct three-dimensional (3D) distributions of transcripts in the mouse brain^53^. Another technology simultaneously monitored DNA and RNA profiles in tissues using SABER protocols^54^. To compute significance of spatial distributions, statistical analysis methods are also being developed for both NGS- and FISH-based technologies^55–57^. These targeted nucleic acid profiling methods and their computational analyses are on the rise for the analysis of tissues from cancer patients. Although transcriptional profiles work robustly in mouse and fresh human specimens, RNA transcripts in archival FFPE patient samples would be relatively more challenging to measure compared with multiplex protein analysis due to the rapid degradation of RNAs over time.

Beyond the central dogma, metabolites also play an important role in the cellular response to therapies. Compared with RNA and protein profiling, it is rather complicated to determine spatially resolved metabolite distributions in native tissues, because metabolites remain noncompatible with amplification methods, the labeling toolkits for metabolites are limited, and the chemical compositions are complex to be deconvolved from mass spectra. Although conventional mass spectrometry imaging (MSI) has provided 5–20 µm resolution for profiling metabolites, recent advancements in instrumentation have allowed acquisition of 1–2 µm spatial features^58^. One such device is atmospheric pressure matrix-assisted laser desorption/ionization (MALDI) that achieved 1.4 µm resolution for imaging metabolites, lipids, and small peptides in single-celled organisms^59^. Another instrument modification was transmission-mode MALDI (t-MALDI-2) that reduced the imaging pixel size to 0.6 µm when recording lipid profiles in mouse organs such as the brain and kidney^60^. A separate instrument was also designed as OrbiSIMS to perform high-resolution imaging of cells and tissues for mapping lipids and neurotransmitters in mouse brain tissues. NanoSIMS, a commercially available device, has also been used to profile metabolites, lipids, and sugars for only seven selected channels at 50–100 nm spatial resolution^61^. Multiplexing metabolites in the same measurement with RNA and protein profiles should impact the current medical practice for cancer diagnosis and treatments.

Simultaneous imaging of RNA and proteins has been implemented in single-cell analysis for mostly in experiments that utilized disassociation of cells and barcoding with nucleic acids^62–66^. Although there is an increasing interest in multiplex RNA and proteins in spatially resolved analysis technologies, only a few transcripts were detected together with multiplex protein maps using mass cytometry and a decent resolution was achieved for simultaneous RNA and protein detection by digital spatial profiling technology^25,67^. Spatial RNA mapping is key to decipher short-time response of transcription activity per gene and spatial protein map is crucial to reach functional state of a cell of interest. Technically, RNA-targeting protocols and protein-staining experiments have distinct needs, making it difficult to unify in a simple experiment. First, the blocking steps include serums such as bovine serum albumin, which degrades the quality of RNA molecules during the washes and treatments. Second, the fixation and permeabilization protocols vary to optimize RNA and protein detection protocols. RNAs require gentle permeabilization methods such as cold ethanol to keep transcripts intact, whereas the proteins need harsh treatments such as methanol and triton that might break RNAs into fragments. RNAs are more sensitive to nuclease activity and RNase might degrade RNA quality; on the other hand, proteins are more robust even in RNase-containing buffers. In addition, metabolite imaging in single cells is highly invasive and the samples are ablated during the image acquisition. Therefore, the ideal spatially resolved multi-omics platform would need to perform RNA targeting “first” and protein staining “second”, and metabolite imaging as the “last” step. Technological and biochemical advances would be required to realize such a truly multi-omics platform for spatial analysis of individual cells.

## Multi-scale bioengineering platforms for spatial dynamics of cells

To understand the molecular pathogenesis, two complementary directions are presented in this perspective, top-down and bottom-up models. The top-down approach targets intact biopsies that maintain the physiological structures of tumors, providing direct representation of the pathology of the tumor. In this direction, tissues are sectioned from solid tumors and CTCs/CTC clusters from liquid biopsies are isolated, followed by visualization of molecular dynamics at the cellular level with multiplex imaging^16^. On the other hand, the bottom-up model utilizes limited types of cell lines (immortal or patient-derived cells) to form a biomimetic tissue environment by culturing monoclonal cells, two-cell types or three-cell types in controlled environments such as artificial gels, microwells, and prototyped microfluidics interfaces. For both models, solid and liquid biopsies show the spatial heterogeneity in different regions of the same tumor or different locations of distinct tumors in the same patient^68^. This perspective will focus on the use of spatial multiplex bioimaging to explore the dynamics and complexity of these top-down and bottom-up approaches as a unified framework to decipher disease mechanisms and progressions.

### Top-down spatial bioengineering

**Fig. 3:**
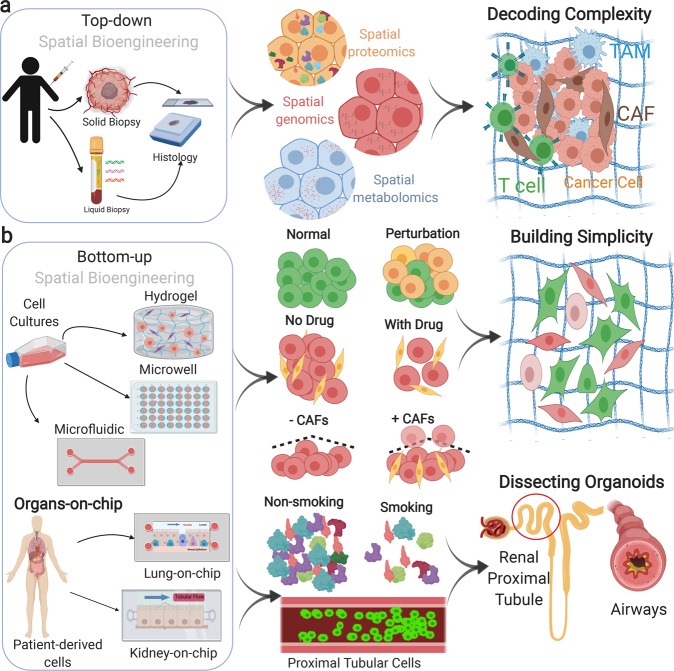
Multi-scale spatial bioengineering of tumors with top-down and bottom-up models. **a** The top-down approach utilizes solid and liquid biopsies that were isolated from patients, followed by fixation, paraffin embedding, and sectioning. Thin tissue specimens were then profiled by spatially resolved imaging methods. Spatial protein profiling in pancreatic cancers using CycIF technology^32^. Spatial genomics analysis of transcriptomes in prostate tumors^39^. Spatial metabolomics analysis of small molecules and lipids in esophageal tissues^58^. **b** Bottom-up research is performed in culture conditions such as hydrogel cultures, microwell molding, and microfluidics-based models. Hydrogel cultures were used to decipher the impact of hypoxia on epithelial–mesenchymal transition in breast cancer cells^123^. Microwell molding was used to quantify the effect of combination chemotherapy on breast cancer cell migration and proliferation^124^. A microfluidic model was used to measure the impact of EGF on breast cancer cell migration^125^. Molecular analysis of organs-on-chip devices was performed. Located in the airway-on-chip device, the time-lapse images showed the ciliary beat frequency of individual cilia in the absence or presence of smoking^126^. The multi-layer microfluidic device, tube-on-chip, comprised the transverse views of a representative hollow fiber section of renal proximal tubule using immunofluorescence staining^92,127,128^. Bottom-up information constructs simplistic interfaces for deciphering tumors and facilitating disease modeling. Created with BioRender.com.

This approach begins with isolating solid or liquid biopsies from patients. Thin tissue specimens are then obtained from tissue blocks that are embedded in preservative chemicals such as paraffin. A microtome is then used to slice the tissue into 5–10 µm sections (Fig. 3a). These tissue slices are then analyzed by multiplex bioimaging techniques. Molecular information is gathered from either tumor biopsies or CTCs. Both of these biopsies reveal genomics, proteomics, and metabolomics markers to study pathogenesis in tumor microenvironments^69^. Although many other efforts utilize ensemble level sequencing and cytometry solutions, the presented work will focus on spatially resolved measurements indirectly sectioned and isolated patient samples at the single-cell level.

Spatial analysis of tissue specimens has classically been performed by immunohistochemistry (IHC) to analyze histopathological features of prognostic factors in distinct tumors. IHC is a colorimetric staining assay based on antibody and color/fluorescence generation chemistry (e.g., horseradish peroxidase) to measure one biomarker at a time on tissues, followed by bright-field imaging and digital analysis of biomarker expression levels on distinct cell types. In breast cancers, prognostic factors measured by IHC remain as traditional histopathological features for predicting patients’ diseases^70,71^. Quantum dots have been used in immunofluorescence (IF) histochemistry (QDs-IHC) to create five-color IHC maps with a brighter signal^72,73^. QD-IHC exhibits more accurate and sensitive results in detecting HER2 in breast cancer than conventional IHC^74^. QDs can be also conjugated with aptamers to target molecules in a single cancer cell^75^. A spectral imaging (Vectra, Perkin Elmer) and Tyramide signal amplification (TSA) scheme have enabled simultaneous eight-color IHC maps in tissue sections from multiple cancers^76^. The same TSA-based reagent labeling was also automated by Ventana for five-color IHC detection in tissues^77^. Multiplex IHC technologies have been implemented up to eight markers so far and clinically have been tested. However, multiplex IHC solutions fail to meet the need for uncovering the complexity of a tumorecosystem that contains up to 50 to a 100 distinct cell types and compositions in diseases.

Another conventional method for analysis of tissue sections is IF to study tumor microenvironment. The microscopic examination enables an understanding of molecular information from tissue to single-cell levels. IF allows measurements for more than three biomarkers using antibody sets that are conjugated to distinct spectral dyes^78^. Although IF has been part of ample clinical and research projects, it is still critical to achieving multiplex IF for more data-driven cellular research in immunology and cancer biology. As the genome is composed of about 20,000 protein-coding genes, ideally multiplex IF should get closer to these multiplexing levels for ultimate profiling solution. Furthermore, antibody-based detection of targets suffers from crosstalk among antibodies due to nonspecific binding events. In practice, multiplexing of IF protocols has detected up to 30–50 protein markers in a single experiment. Cyclic labeling and fluorescence imaging methods^30,31,33,79,80^ (CODEX, CycIF, MxIF, and Immuno-SABER) and one-shot MSI of isotope labeling techniques^20,35^ (IMC and MIBI) have delivered this premise of measuring up to 56 markers in FFPE tissue sections. For instance, tissue-optimized CycIF profiled a 24-marker panel in patient specimens from pancreatic cancers, providing anti-correlated single-cell distributions of E-cadherin and Vimentin^32^. These spatially resolved protein-imaging methods are the initial efforts for a spatial proteomics vision^81^ that impacts next-generation histological analysis.

Alternative spatially resolved profiling methods can be applied to the top-down analysis. Spatial genomics and transcriptomics technologies with genome-scale mapping (ST, HDST, and others in Table 1) and with targeted mapping (STARMAP, seqFISH, MERFISH, and others in Table 1) provide gene expression measurements in tissues as a molecular activity. For instance, in situ sequencing of amplified signals of individual transcripts was profiled in breast cancer tissues using direct hybridization of targeted markers^50^. Spatial maps of prostate cancers were also generated based on ST technology^39^. Complementarily, spatial metabolomics approaches also provided region-specific small molecule and lipid profiles by mass spectrometry analysis in esophageal tissues^58^. Instrumental advances^59,60,82^ in MSI methods (MALDI and SIMS) will be applicable to human tissues to distinguish between health and disease form metabolic alterations at sub-micrometer resolution levels.

### Top-down cellular dynamics

Although in vivo research could provide physiological information about the effects of drugs and mechanisms directly from patients’ responses, molecular underpinnings can be studied more extensively and produce more reproducible data by controlling and modifying the cultured cells after the live specimens are isolated from humans. Ex vivo model incorporates the advantages of both in vivo and in vitro models. As ex vivo research is based on tissue biopsies, the architecture of the cells is maintained to preserve the in vivo conditions^16^. In the meantime, in vitro models are based on cultured cells that can be used for systematic dissection of dynamic cellular interactions in controlled experimental conditions. For example, isolating tumor-cytolytic T cells directly from the patient’s bloodstream maintains the cytolytic potential and provide physiologically relevant samples for studying adoptive cellular immunotherapy after ex vivo expansion^83^. Experiments with 3D ex vivo models recapitulate tumorigenesis from early genetic events before phenotypic changes. Furthermore, ex vivo models have the potential to study the individual cellular and humoral component contributing to the tumorigenesis each at a time^84^. Therefore, ex vivo platforms can be interfaced with imaging techniques to identify the spatial and epigenetic changes from the subcellular level under an experimental condition that is similar to the in vivo molecular and cellular states. Multiplex bioimaging methods are well-suited to study ex vivo platforms for deciphering dynamics of combinatorial drug treatments and their molecular response in the RNA, protein, and metabolite levels at the single-cell level.

### Bottom-up spatial bioengineering

Tissue engineering field has adopted bioengineering platforms for recapitulating clinically relevant biophysical and the biochemical crosstalk between cells and their extracellular environment. One common approach is the “bottom-up”, in which tissues are fabricated with a few types of cells to resemble the microarchitecture features of the native tissue. Bottom-up platforms were designed by several techniques that include culturing cells in hydrogels, molded microwells, and microfluidic models. Controlled cell aggregation is the most common technique that is used to create biomimetic modular tissues with a specific microarchitecture^85^. Microscale models of tissues can then be converted to macroscale biomimetic models of tissues and organs using advanced technologies such as tissue printing, cell-sheet fabrication, and cell-laden hydrogels fabrication^86^. Bottom-up models provide insightful information about the cellular interaction and tissue compositions, but they exhibit shortcomings due to the limited cellular types that are used in the model, and the variant mechanical and structural properties. For example, an in vitro breast cancer model was developed on a chip that mimicked the interaction between several factors of the extracellular matrix (ECM) with the breast cancer cells (Fig. 3b). This model was used to study drug perturbation of a nanoparticle-based drug delivery system. This microfluidic-based model allowed the evaluation of drug transport and its cytotoxic effects in engineered microscale conditions^87^. Multiplex bioimaging can be interfaced with these engineered microsystems to systematically understand downstream and upstream molecular drivers of two-cell or three-cell interactions. Molecular changes of monoclonal cell lines also can be elucidated based on multiplex imaging profiles of specific gene sets that differentially change on the micro-engineered cellular platforms.

### Bottom-up dissection of organs-on-chips

Designing improved cancer diagnostic tools and therapeutic agents require a better understanding of the complex interaction between cancer cells and the surrounding microenvironment. To address this challenge, organ-on-chip models are used to recapitulate the intricate multicellular architecture of human cancers. Organs-on-chips are microfluidic, multichannel cell culture chips, and they contain several hallow channels with distinct organizations to simulate the biochemical and the biophysical features of diseases at the organ level (Fig. 3b)^88^. For example, a human-based in vitro lung cancer model was developed to study the process of tumor growth in response to the mechanical stress/strain of breathing. The process of breathing was modeled to determine mechanically active tumor models. Distinct cell invasion and drug response profiles revealed that the tumor microenvironment can significantly influence cancerogenesis^89^. Organs-on-chips are superior to conventional bottom-up approaches, as it recapitulates more features related to tissue architecture and multicellular interaction. Organ-on-chip can also benefit from the power of multiplexed imaging to visualize spatial maps of cells organization and to analyze the distribution of stromal cells in the TMEs.

### Spatial bottom-up analysis of cell-based therapies

**Fig. 4:**
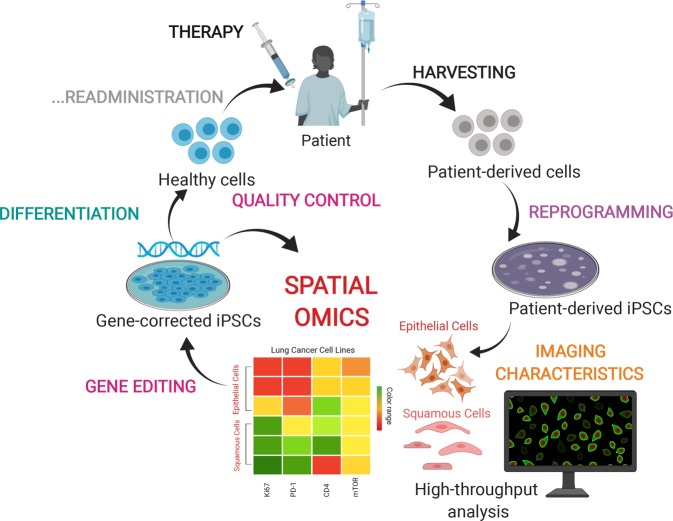
Applying multiplex spatial imaging to patient-derived cell therapy. Spatially resolved omics profiling can also be applied to the process of cell/gene therapy. Patient-derived cells are harvested and reprogrammed to iPSCs in vitro conditions. By screening the spatial molecular maps of the iPSCs before (discovery) and after (quality control) gene-editing the cells, quantitative and bioinformatics analysis can identify the efficacy of the targeted drugs in patients and determine heterogeneity across iPSC populations. iPSCs can then be engineered and differentiated to the healthy phenotypes. Created with BioRender.com.

Another revolutionary approach in the field of tissue engineering and precision medicine is cell-based therapies. In this technique, intact living cells are harvested from patients or donors to be modified within in vitro conditions (Fig. 4). The engineered cells can then be implanted, injected, or grafted into patients to cure a pathological condition. This approach has many promising applications including fighting cancers through the cell-mediated immunotherapies and addressing cardiovascular and musculoskeletal conditions. However, this technique suffers from several risks associated with disease transmission, heterogeneity of the implanted cellular materials, and immune rejection. Routine imaging techniques are used to detect few makers associated with severe immune responses, but that is insufficient to ensure the safety and the efficacy of cell-based therapies^90^. Thereby, cell-based therapies can greatly benefit from multiplexed imaging techniques to detect many markers associated with cellular heterogeneity, ensuring the safety of cellular implantation.

### Complementary domains of top-down and bottom-up approaches

Top-down research approach is excellent in capturing the tissue complexity in its native conditions as presented in Table 2. However, it is challenging to get reproducible data from the same sampling site or from the same donor due to the heterogeneity of disease pathology among patients, and even in the same patient. As top-down research approaches capture native tissues from patients’ biopsies, it produces a myriad of information about the proteomic and the genetic profiles, which can lead to discoveries of new biomarkers associated with diseases or potential targets for drugs. Given the dynamic nature of human diseases, it is challenging to get biopsies at all stages of a disease, which makes it challenging to make definitive conclusions about disease progression. Top-down research yields results with higher clinical relevance, but getting human biopsies can be expensive, invasive, and ethically controversial. On the other hand, bottom-up research approaches study biological behaviors within well-studied, controlled conditions, which makes it easier to analyze the data and to draw conclusions. Using a limited number of variables also makes it possible to discover the interdependence of biological behaviors. Nonetheless, bottom-up research approaches can oversimplify biological processes, which make it challenging to capture patients’ heterogeneity and to transfer data to clinical settings.

**Table 2:** Multi-scale spatial bioengineering. Comparisons of the “Top-down” and “Bottom-up” research approaches that are integrated with multiplexing bioimaging technologies.

	Top-down advantages	Top-down drawbacks	Bottom-up advantages	Bottom-up drawbacks
Complexity	Intact tissues and many cell types	Intricate cellular analysis	Systematic study of cell interactions	Limited number of cell types
Dynamics	Biomarker discovery	Challenges to collect tissue biopsies	Discover responses of controlled cell groups	Lacks patient-related heterogeneity
Clinical	Direct medical use	High cost, ethical, invasive procedures	Generation of reproducible data	Difficult to transfer to clinical practice

## Precision medicine by spatial multi-omics and artificial intelligence

Personalized medicine has been a long-lasting interest for clinicians and researchers for many years^91^. Precision therapies for a particular patient at the right time is the definition of personalized medicine that dates back to Hippocrates^92^. In the post-genomic era, Human Genome Project elucidated an individual’s genomic profile at a deeper molecular level in 2001 and NGS has enabled rapid genome-wide mapping of patient’s samples at low cost^93^. In 2008, officials initiated regulatory efforts on the nationwide need for personalized medicine to address the costs and quality of life associated with critical diseases including cancers^94^. These personalized health initiatives have become an integral part of the National Institute of Health to enable innovative oncology treatment options based on an emerging “cancer knowledge network” that can then be expanded to other diseases such as chronic conditions^95^. Awareness of precision medicine for a clinical model that integrates genomics profiles and patient-related information to improve health outcomes is on the rise^96^.

Multi-omics approaches in personalized medicine have become the recent paradigm in medical practice. Although the advances in genome sequencing have piqued the interest for individualized treatments, other molecular profiling methods to measure the epigenome, transcriptome, proteome, and metabolome, along with environmental factors, have impacted clinical decision-making^97^. Technological advances in instruments and computational approaches have made it possible to combine multi-omics profiles into an integrated metric for defining health and disease^98^. These multiparameter patient profiles routinely create big data that allows highly specific patient stratification, biomarker discovery, and functional drug kinetics^99^. Patient subtyping lies at the heart of personalized treatments^100^. For a specific treatment plan, patients either respond or resist chemotherapy, small molecule therapy (hormone and signaling), and immunotherapies. These patients are mainly classified as “responders” and “non-responders,” which are then used as part of the disease knowledge libraries. This classification can further be converted to decision trees based on the molecular and characteristic information of the individual patients.

**Fig. 5:**
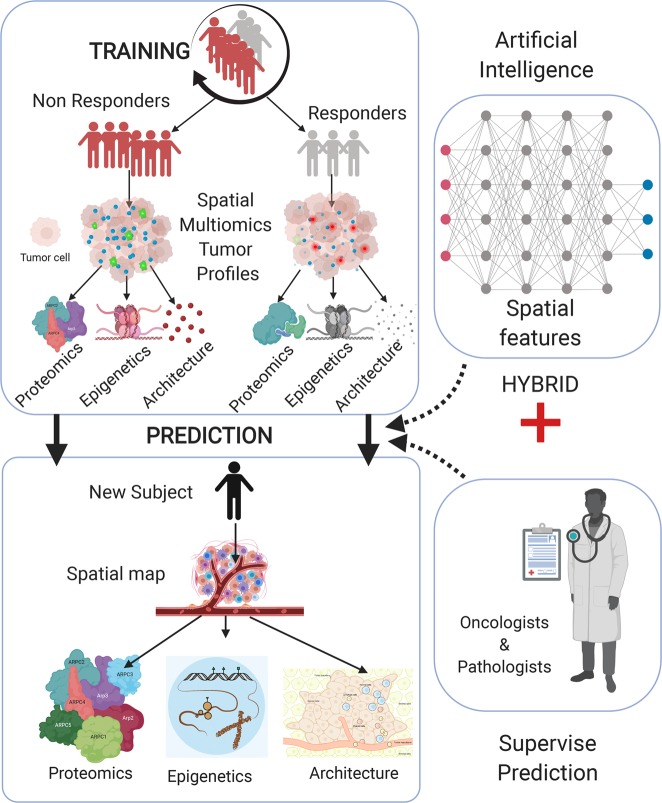
Precision oncology with artificial intelligence using spatial multi-omics. Patient biopsies are profiled by spatial bioimaging technologies that integrate proteomic, genomic, epigenetic, and architectural (structural and cell groups) data for analysis of tumor compositions. The therapeutic response or resistance of individual subjects is then used to classify the patient subgroups. An artificial intelligence framework leverages patient characteristics and spatial multi-omics profiles as training datasets. Spatial features comprising cellular positions, interaction frequencies, tissue structural variations, and subcellular variations are included in the machine-learning analysis. The treatment plan for a new subject will be predicted based on the training set and will be supervised by oncologists and pathologists for accuracy. Personalized doses of radio/chemo/immunotherapies and combination drugs will be tested and validated in next-generation clinical trials. Created with BioRender.com.

Most of the multi-omics studies in clinics are based on conventional ensemble level measurements. However, cancers are regulated by the cellular interactions in tumors, wherein the subcellular responses determine personalized treatment options. Single-cell sequencing and single-cell mass cytometry methods have provided disease-associated cellular phenotypes and their abundances in various cancers^101,102^. These methods utilize cellular suspensions from patient-derived blood and sorted cells to study the drug resistance in therapies. Single-cell variations uncovered the principles of tumor compositions and specific immune subtypes that differentially change in distinct cancers. For instance, natural killer cells showed the cytolytic activity in lung adenocarcinoma, an emerging cellular phenotype for immunotherapies^103^. These single-cell methods still lack one more dimension, the spatial extent of cellular interactions^101,104^. Spatial-omics technologies that are presented in this perspective are ideal platforms for designing personalized treatments. Single-cell phenotypes and their local interactions are both captured in spatially resolved proteomics, genomics, and metabolomics technologies in patient specimens. Spatial multi-omics approaches will be the ultimate molecular profiles of resistant and response patient groups, together with patient characteristics (Fig. 5).

Artificial intelligence is a branch of computer science that develops and applies advanced mathematical and computational approaches to “learn” the complex clinical data structure and to “predict” a therapeutic metric from that data set based on measurable features, providing ample opportunities for precision medicine^105^. Patient stratification is an important aspect of personalized therapies to model the complex heterogeneity of individuals. Machine-learning and deep-learning frameworks have enabled subtyping of patients based on molecular data (genomics, proteomics, and metabolomics), pathological maps, and molecular imaging data^106,107^. Although these datasets are from the same patients, there is still a challenge of combining these multi-omics data with different characteristics. The performance of patient classifications suffers from these incompatible data types^105^. To address these issues, spatial multi-omics approaches offer high sensitivity and direct visualization of transcript, protein, and metabolite profiles that will potentially improve the patient stratification. Spatial profiles are well-aligned at the cellular level, making them consistent across individual patients or across patient cohorts. Another significant challenge for machine learning in medicine is the size of the datasets. Although genome-level sequencing results are valuable, spatial single-cell data generates massive molecular maps at the subcellular and single-cell levels. This cellular big data generated by the spatially resolved analyses will then enhance the performance of automated stratification of patient groups. Generated from spatial multi-omics platforms, smart patient classifications can then be integrated with drug response to a given therapy. This comprehensive information about patient groups (responder vs. non-responders) and correlation to molecular subtypes of patients (proteomics, epigenetics, spatial maps, and other targets) will serve as “training” of the machine- and deep-learning algorithms, initial step of the precision oncology framework (Fig. 5).

Using artificial intelligence solutions, prediction of patients’ treatment outcome has been applied to imaging data and molecular maps in particularly radiology and pathology^108^. Response to neoadjuvant therapy was predicted from pretreatment PET images with quantitative features that were analyzed by convolutional neural networks in esophageal cancers^109^. Survival patterns of patients were predicted from spatial distance measurements in MRI images from subjects with glioblastoma multiforme tumors^110^. The likelihood of recurrence was also predicted from a classifier algorithm that quantified tumor-infiltrating lymphocytes in pathology images^111^. An integration of pathological spatial features and molecular genome-wide data in thousands of patients allowed predictions of clinical outcomes^112^. Further, multiplexed single-cell proteomic profiles were measured in pediatric diseases and a machine-learning model (regularized elastic-net approach) was used to incorporate cellular developmental aberrations to predict the relapse of leukemias^113^. Similarly, a subset of glioblastoma cells were identified as the response predicter from single-cell signaling profiles without the need for prior information about the expected cell clusters on bioinformatics maps^114^. When combined with spatial multi-omics technologies, bioimaging features (cellular positions, interaction frequencies, tissue structural variations, and subcellular distributions) from single-cell analysis and molecular imaging will continue impacting predictive outcome studies in precision medicine.

Another benefit of the machine-learning algorithm is to design a personalized dose for therapy. A deep profiler framework was developed using pretreatment CT scan images that identified radiotherapy dose requirements for individual patients^115^. The 3D dose–response in 120 patients with head and neck cancers was predicted by a deeply connected U-net method, providing accurate 3D distributions of the radiotherapies in the head and neck regions^116^. In addition to personalized dose design, artificial intelligence aids the development of combinatorial drug treatments. Tumor cell lines were studied in response to a high-throughput drug screening assay, wherein a deep neural network model showed higher efficacy for more than two-drug combinations. Synergistic combinations of drugs were also explored by a DeepSynergy algorithm that utilized machine learning on experimental and clinically approved drugs on cell cultures^117^. The presented framework in this paper that combine top-down and bottom-up approaches can benefit from personalized dose predictions on multiplex histological analysis from patient biopsies, and at the same time, combinatorial drug treatments can be optimized by spatial-omics maps on high-throughput cell cultures to reveal synergistic drug groups in cancer treatments.

The prediction model in spatial multi-omics data can greatly enhance the accuracy and sensitivity compared with the classical omics data as the dynamic range of cellular phenotypes and their corresponding functional differences can be mapped out with finer details. The rare and dominant cellular subgroups can be classified into two distinct clusters in the prediction to balance the dynamic range differences of heterogeneous cellular populations. In other words, the average spatial multi-omics data already contains information that would be captured by conventional sequencing approaches, but now also with additional sub-composition information as an additive parameter in the model. The larger and the more intricate the input data is, the finer the predictions will be for precision oncology. For instance, in a supervised prediction model, the spatial extent of biopsies can be calculated to define immune- and tumor-enriched regions. These larger-sized tissue architectures can then be separated into cellular distributions for a given area. For each cell, subcellular variations can be computed to further regularize predictions. Of note, these hierarchical structures are connected and reach the full extent of biopsy samples at different spatial details. Briefly, the tissue neighbors would show data that is split into, e.g., 10 × 10 regions. The single-cell distributions would split into 100 × 100 spatial features and subcellular differences would further classify the same tissue to 1000 × 1000 data points. A supervised model can then use this well-defined data structure to output consistent predictions for personalized therapies.

The projected use of artificial intelligence algorithms for modeling spatial multi-omics data is feasible based on spatial imaging features such as 3D distributions, lateral and axial dimensions, orientation in 3D space, and encoded colors^118^. However, it necessitates stringent processing conditions for robustness and performance comparisons with classical machine-learning algorithms such as support vector machines and random forest trees for validation^119^. Comparative analysis of complex models^120^ from conventional multi-omics data and computational predictions^121^ from emerging spatial multi-omics data would be one of the central debates for next-generation precision oncology contexts.

Finally, spatial features from tissue compositions, cellular positions, and their relative distributions, and subcellular heterogeneity would need a “reference framework” to construct relative spatial maps for each individual patient. Previously, a dynamic immune system was modeled by a “scaffold” framework to define reproducible cell populations in mouse and human immune systems across various organs^122^. Such a reference map handles high-dimensional mass cytometry data from single-cell details. The relative positioning of immune-cancer cells from fine pathological data can be incorporated into landmarks, creating a spatial reference framework per patient. The cellular neighborhood approach also was introduced in the CODEX proteomic analysis to define normal and diseased spleen architecture from multiparameter single-cell maps^33^. Spatial multi-omics datasets can use a hybrid model to compute hierarchical neighborhoods and a reference framework to capture patient-to-patient variations. Graphical representations and network biology would be the single-cell route to visualize and handle this spatial complexity without overfitting issues.

## Conclusions

Tumors are complex ecosystems that are composed of multiple cell types interacting with each other. Cancer cells respond to therapies under the effect of other cell types in the tumor microenvironment. To decipher heterogeneity of drug treatments in distinct patients, cellular compositions need comprehensive molecular characterization at the single-cell level. During the metastasis, these cellular types leak into the bloodstream until they reach a secondary location. Through liquid biopsies, analysis of clustered cells and isolated cells in the bloodstream also provides complementary information about the patient’s phenotype and potential for therapeutic design. To this end, spatially resolved bioimaging technologies reveal genomic, proteomic, and metabolic variability of individual cells in both solid and liquid biopsy samples. This approach provides a top-down approach to study disease progression.

To recapitulate the heterogeneity of tumor microenvironment, an increasing number of approaches have been developed to mimic patient samples in artificial on-chip environments. Tissue-engineered platforms and organs-on-chip are designed to study cellular interactions with a controlled in vitro model to understand one-cell, two-cell, or multi-cell groups for monitoring dynamics and combinatorial drug treatments. Multiplex bioimaging methods can elucidate upstream and downstream factors in these bottom-up bioengineering platforms, providing complementary information about the molecular profiles and response characteristics of patient-derived cells.

Multiple biomarkers in the tumor, cell cluster, and subcellular microenvironment will be measured in a group of patients who respond or resist therapies. An artificial intelligence method such as a machine or deep learning will then use these spatial multi-omics maps with significant features as training datasets. Learning-based predictions will then make it possible to group and personalize the new patient’s treatment according to the spatial-omics information that associates the physical, genetic, and biochemical properties of the tumors. The presented AI-based high-dimensional histological analysis pipeline will be validated in clinical trials with expected outcomes and matching predictive scores, demonstrating the use of a precision medicine approach and beyond.

## Data Availability

Any display item and related data are available upon request.

## References

[1] Evan GI, Vousden KH (2001). Proliferation, cell cycle and apoptosis in cancer. Nature.

[2] Fisher R, Pusztai L, Swanton C (2013). Cancer heterogeneity: implications for targeted therapeutics. Br. J. Cancer.

[3] Mitrus I, Bryndza E, Sochanik A, Szala S (2012). Evolving models of tumor origin and progression. Tumour Biol..

[4] Reya T, Morrison SJ, Clarke MF, Weissman IL (2001). Stem cells, cancer, and cancer stem cells. Nature.

[5] Mitra A, Mishra L, Li S (2015). EMT, CTCs and CSCs in tumor relapse and drug-resistance. Oncotarget.

[6] Shaffer SM (2017). Rare cell variability and drug-induced reprogramming as a mode of cancer drug resistance. Nature.

[7] Garraway LA, Jänne PA (2012). Circumventing cancer drug resistance in the era of personalized medicine. Cancer Discov..

[8] Friedman AA, Letai A, Fisher DE, Flaherty KT (2015). Precision medicine for cancer with next-generation functional diagnostics. Nat. Rev. Cancer.

[9] Vogelstein B, Kinzler KW (2004). Cancer genes and the pathways they control. Nat. Med..

[10] Hocking J, Mithraprabhu S, Kalff A, Spencer A (2016). Liquid biopsies for liquid tumors: emerging potential of circulating free nucleic acid evaluation for the management of hematologic malignancies. Cancer Biol. Med..

[11] Jahr S (2001). DNA fragments in the blood plasma of cancer patients: quantitations and evidence for their origin from apoptotic and necrotic cells. Cancer Res..

[12] Kurtz DM (2018). Circulating tumor DNA measurements as early outcome predictors in diffuse large B-cell lymphoma. JCO.

[13] De Vlaminck I (2014). Circulating cell-free DNA enables noninvasive diagnosis of heart transplant rejection. Sci. Transl. Med..

[14] Hofman P, Heeke S, Alix-Panabières C, Pantel K (2019). Liquid biopsy in the era of immuno-oncology: is it ready for prime-time use for cancer patients?. Ann. Oncol..

[15] Alix-Panabières C, Pantel K (2013). Circulating tumor cells: liquid biopsy of cancer. Clin. Chem..

[16] Dusinska, M., Rundén-Pran, E., Schnekenburger, J. & Kanno, J. in *Adverse Effects of Engineered Nanomaterials* (eds Fadeel, B., Pietroiusti, A. & Shvedova, A. A.) 2nd edn, 51–82 10.1016/B978-0-12-809199-9.00003-3 (Academic Press, 2017).

[17] Frangioni JV (2008). New technologies for human cancer imaging. J. Clin. Oncol..

[18] Vaquero JJ, Kinahan P (2015). Positron emission tomography: current challenges and opportunities for technological advances in clinical and preclinical imaging systems. Annu Rev. Biomed. Eng..

[19] Altorki NK (2019). The lung microenvironment: an important regulator of tumour growth and metastasis. Nat. Rev. Cancer.

[20] Keren L (2019). MIBI-TOF: a multiplexed imaging platform relates cellular phenotypes and tissue structure. Sci. Adv..

[21] Dasgupta A, Lim AR, Ghajar CM (2017). Circulating and disseminated tumor cells: harbingers or initiators of metastasis?. Mol. Oncol..

[22] Adams DL (2015). Cytometric characterization of circulating tumor cells captured by microfiltration and their correlation to the CellSearch(®) CTC test. Cytom. A.

[23] Sarioglu AF (2015). A microfluidic device for label-free, physical capture of circulating tumor cell clusters. Nat. Methods.

[24] Giesen C (2014). Highly multiplexed imaging of tumor tissues with subcellular resolution by mass cytometry. Nat. Methods.

[25] Schulz D (2018). Simultaneous multiplexed imaging of mRNA and proteins with subcellular resolution in breast cancer tissue samples by mass cytometry. Cell Syst..

[26] Gerdtsson E (2018). Multiplex protein detection on circulating tumor cells from liquid biopsies using imaging mass cytometry. Converg. Sci. Phys. Oncol.

[27] Payne RE (2012). Viable circulating tumour cell detection using multiplex RNA in situ hybridisation predicts progression-free survival in metastatic breast cancer patients. Br. J. Cancer.

[28] Boettiger AN (2016). Super-resolution imaging reveals distinct chromatin folding for different epigenetic states. Nature.

[29] Xu J (2018). Super-resolution imaging of higher-order chromatin structures at different epigenomic states in single mammalian cells. Cell Rep..

[30] Saka SK (2019). Immuno-SABER enables highly multiplexed and amplified protein imaging in tissues. Nat. Biotechnol..

[31] Lin J-R, Fallahi-Sichani M, Sorger PK (2015). Highly multiplexed imaging of single cells using a high-throughput cyclic immunofluorescence method. Nat. Commun..

[32] Lin J-R (2018). Highly multiplexed immunofluorescence imaging of human tissues and tumors using t-CyCIF and conventional optical microscopes. eLife.

[33] Goltsev Y (2018). Deep profiling of mouse splenic architecture with CODEX multiplexed imaging. Cell.

[34] Bendall SC (2011). Single-cell mass cytometry of differential immune and drug responses across a human hematopoietic continuum. Science.

[35] Jackson HW (2020). The single-cell pathology landscape of breast cancer. Nature.

[36] Keren L (2018). A structured tumor-immune microenvironment in triple negative breast cancer revealed by multiplexed ion beam imaging. Cell.

[37] Ståhl PL (2016). Visualization and analysis of gene expression in tissue sections by spatial transcriptomics. Science.

[38] Moncada R (2020). Integrating microarray-based spatial transcriptomics and single-cell RNA-seq reveals tissue architecture in pancreatic ductal adenocarcinomas. Nat. Biotechnol.

[39] Berglund E (2018). Spatial maps of prostate cancer transcriptomes reveal an unexplored landscape of heterogeneity. Nat. Commun..

[40] Rodriques SG (2019). Slide-seq: a scalable technology for measuring genome-wide expression at high spatial resolution. Science.

[41] Vickovic S (2019). High-definition spatial transcriptomics for in situ tissue profiling. Nat. Methods.

[42] Lee JH (2014). Highly multiplexed subcellular RNA sequencing in situ. Science.

[43] Weinstein JA, Regev A, Zhang F (2019). DNA microscopy: optics-free spatio-genetic imaging by a stand-alone chemical reaction. Cell.

[44] Lubeck E, Coskun AF, Zhiyentayev T, Ahmad M, Cai L (2014). Single-cell in situ RNA profiling by sequential hybridization. Nat. Methods.

[45] Coskun AF, Cai L (2016). Dense transcript profiling in single cells by image correlation decoding. Nat. Methods.

[46] Eng C-HL (2019). Transcriptome-scale super-resolved imaging in tissues by RNA seqFISH. Nature.

[47] Shah S (2018). Dynamics and spatial genomics of the nascent transcriptome by intron seqFISH. Cell.

[48] Chen KH, Boettiger AN, Moffitt JR, Wang S, Zhuang X (2015). Spatially resolved, highly multiplexed RNA profiling in single cells. Science.

[49] Xia C, Fan J, Emanuel G, Hao J, Zhuang X (2019). Spatial transcriptome profiling by MERFISH reveals subcellular RNA compartmentalization and cell cycle-dependent gene expression. PNAS.

[50] Ke R (2013). In situ sequencing for RNA analysis in preserved tissue and cells. Nat. Methods.

[51] Shah S (2016). Single-molecule RNA detection at depth by hybridization chain reaction and tissue hydrogel embedding and clearing. Development.

[52] Sylwestrak EL, Rajasethupathy P, Wright MA, Jaffe A, Deisseroth K (2016). Multiplexed intact-tissue transcriptional analysis at cellular resolution. Cell.

[53] Wang X (2018). Three-dimensional intact-tissue sequencing of single-cell transcriptional states. Science.

[54] Kishi JY (2019). SABER amplifies FISH: enhanced multiplexed imaging of RNA and DNA in cells and tissues. Nat. Methods.

[55] Edsgärd D, Johnsson P, Sandberg R (2018). Identification of spatial expression trends in single-cell gene expression data. Nat. Methods.

[56] Sun S, Zhu J, Zhou X (2020). Statistical analysis of spatial expression patterns for spatially resolved transcriptomic studies. Nat. Methods.

[57] Zhu, Q., Shah, S., Dries, R., Cai, L. & Yuan, G.-C. Identification of spatially associated subpopulations by combining scRNA-seq and sequential fluorescence in situ hybridization data. *Nat. Biotechnol*. 10.1038/nbt.4260 (2018). 10.1038/nbt.4260 PMC6488461 30371680

[58] Sun C (2019). Spatially resolved metabolomics to discover tumor-associated metabolic alterations. PNAS.

[59] Kompauer M, Heiles S, Spengler B (2017). Atmospheric pressure MALDI mass spectrometry imaging of tissues and cells at 1.4-μm lateral resolution. Nat. Methods.

[60] Niehaus M, Soltwisch J, Belov ME, Dreisewerd K (2019). Transmission-mode MALDI-2 mass spectrometry imaging of cells and tissues at subcellular resolution. Nat. Methods.

[61] He C (2018). NanoSIMS analysis of intravascular lipolysis and lipid movement across capillaries and into cardiomyocytes. Cell Metab..

[62] Darmanis S (2016). Simultaneous multiplexed measurement of RNA and proteins in single cells. Cell Rep..

[63] Frei AP (2016). Highly multiplexed simultaneous detection of RNAs and proteins in single cells. Nat. Meth.

[64] Albayrak C (2016). Digital quantification of proteins and mRNA in single mammalian cells. Mol. Cell.

[65] Lin J (2019). Ultra-sensitive digital quantification of proteins and mRNA in single cells. Nat. Commun..

[66] Cheow LF (2016). Single-cell multimodal profiling reveals cellular epigenetic heterogeneity. Nat. Methods.

[67] Beechem, J. M. in *Biomarkers for Immunotherapy of Cancer: Methods and Protocols* (eds Thurin, M., Cesano, A. & Marincola, F. M.) 563–583 10.1007/978-1-4939-9773-2_25 (Springer New York, 2020).

[68] Ilié M, Hofman P (2016). Pros: can tissue biopsy be replaced by liquid biopsy?. Transl. Lung Cancer Res.

[69] Chaurand P, Sanders ME, Jensen RA, Caprioli RM (2004). Proteomics in diagnostic pathology: profiling and imaging proteins directly in tissue sections. Am. J. Pathol..

[70] Elston CW, Ellis IO (1991). Pathological prognostic factors in breast cancer. I. The value of histological grade in breast cancer: experience from a large study with long-term follow-up. Histopathology.

[71] Cireşan, D. C., Giusti, A., Gambardella, L. M. & Schmidhuber, J. in *Medical Image Computing and Computer-Assisted Intervention – MICCAI 2013* (eds Mori, K., Sakuma, I., Sato, Y., Barillot, C. & Navab, N.) 411–418 (Springer, Berlin, 2013).

[72] Chen H (2009). Comparison of quantum dots immunofluorescence histochemistry and conventional immunohistochemistry for the detection of caveolin-1 and PCNA in the lung cancer tissue microarray. J. Mol. Hist..

[73] Xing Y (2007). Bioconjugated quantum dots for multiplexed and quantitative immunohistochemistry. Nat. Protoc..

[74] Chen C (2009). Quantum dots-based immunofluorescence technology for the quantitative determination of HER2 expression in breast cancer. Biomaterials.

[75] Kang WJ, Chae JR, Cho YL, Lee JD, Kim S (2009). Multiplex imaging of single tumor cells using quantum-dot-conjugated aptamers. Small.

[76] Gorris MAJ (2018). Eight-color multiplex immunohistochemistry for simultaneous detection of multiple immune checkpoint molecules within the tumor microenvironment. J. Immunol..

[77] Zhang W (2017). Fully automated 5-plex fluorescent immunohistochemistry with tyramide signal amplification and same species antibodies. Lab. Invest..

[78] Najjar YG (2019). Tumor cell oxidative metabolism as a barrier to PD-1 blockade immunotherapy in melanoma. JCI Insight.

[79] Schürch, C. M. et al. Coordinated cellular neighborhoods orchestrate antitumoral immunity at the colorectal cancer invasive front. Preprint at bioRxiv 743989 10.1101/743989 (2019).

[80] McKinley ET (2017). Optimized multiplex immunofluorescence single-cell analysis reveals tuft cell heterogeneity. JCI Insight.

[81] Lundberg E, Borner GHH (2019). Spatial proteomics: a powerful discovery tool for cell biology. Nat. Rev. Mol. Cell Biol..

[82] Passarelli MK (2017). The 3D OrbiSIMS—label-free metabolic imaging with subcellular lateral resolution and high mass-resolving power. Nat. Methods.

[83] Rubio V (2003). Ex vivo identification, isolation and analysis of tumor-cytolytic T cells. Nat. Med..

[84] Wulf G, Garg P, Liou Y-C, Iglehart D, Lu KP (2004). Modeling breast cancer in vivo and ex vivo reveals an essential role of Pin1 in tumorigenesis. EMBO J..

[85] Dean DM, Napolitano AP, Youssef J, Morgan JR (2007). Rods, tori, and honeycombs: the directed self-assembly of microtissues with prescribed microscale geometries. FASEB J..

[86] Nichol JW, Khademhosseini A (2009). Modular tissue engineering: engineering biological tissues from the bottom up. Soft Matter.

[87] Chen Y, Gao D, Wang Y, Lin S, Jiang Y (2018). A novel 3D breast-cancer-on-chip platform for therapeutic evaluation of drug delivery systems. Anal. Chim. Acta.

[88] Sontheimer-Phelps A, Hassell BA, Ingber DE (2019). Modelling cancer in microfluidic human organs-on-chips. Nat. Rev. Cancer.

[89] Hassell BA (2018). Human organ chip models recapitulate orthotopic lung cancer growth, therapeutic responses, and tumor dormancy in vitro. Cell Rep..

[90] Alvi, K. Cell culture technology for pharmaceutical and cell-based therapies. Edited by S. S. Ozturk and W.-S. Hu. CRC Press/Taylor & Francis, Boca Raton. *J. Nat. Prod*. **70**, 712–713 https://pubs.acs.org/doi/full/10.1021/np078140a (2006).

[91] Offit K (2011). Personalized medicine: new genomics, old lessons. Hum. Genet..

[92] Steele FR (2008). Personalized medicine: something old, something new. Personalized Med..

[93] Carrasco-Ramiro F, Peiró-Pastor R, Aguado B (2017). Human genomics projects and precision medicine. Gene Ther..

[94] Wells RC (2009). A new President, a new Congress and the path to personalized medicine. Personalized Med..

[95] Collins FS, Varmus H (2015). A new initiative on precision medicine. N. Engl. J. Med..

[96] Pritchard DE (2017). Strategies for integrating personalized medicine into healthcare practice. Personalized Med..

[97] Chen R, Snyder M (2013). Promise of personalized omics to precision medicine. Wiley Interdiscip. Rev. Syst. Biol. Med..

[98] Huang S, Chaudhary K, Garmire LX (2017). More is better: recent progress in multi-omics data integration methods. Front. Genet.

[99] Gligorijević V, Malod-Dognin N, Pržulj N (2016). Integrative methods for analyzing big data in precision medicine. Proteomics.

[100] Wang D, Gu J (2016). Integrative clustering methods of multi-omics data for molecule-based cancer classifications. Quant. Biol..

[101] Shalek AK, Benson M (2017). Single-cell analyses to tailor treatments. Sci. Transl. Med..

[102] Galli E (2019). The end of omics? High dimensional single cell analysis in precision medicine. Eur. J. Immunol..

[103] Lavin Y (2017). Innate immune landscape in early lung adenocarcinoma by paired single-cell analyses. Cell.

[104] Regev A (2017). The Human Cell Atlas. eLife.

[105] Azuaje F (2019). Artificial intelligence for precision oncology: beyond patient stratification. npj Precis. Onc.

[106] Valdes G (2016). MediBoost: a patient stratification tool for interpretable decision making in the era of precision medicine. Sci. Rep..

[107] Gao F (2019). DeepCC: a novel deep learning-based framework for cancer molecular subtype classification. Oncogenesis.

[108] Lambin P (2013). Predicting outcomes in radiation oncology—multifactorial decision support systems. Nat. Rev. Clin. Oncol..

[109] Ypsilantis P-P (2015). Predicting response to neoadjuvant chemotherapy with PET imaging using convolutional neural networks. PLOS ONE.

[110] Zhou, M., Hall, L. O., Goldgof, D. B., Gillies, R. J. & Gatenby, R. A. in *Medical Imaging 2013: Computer-Aided Diagnosis*. vol. 8670 86702O (International Society for Optics and Photonics, 2013).

[111] Corredor G (2019). Spatial architecture and arrangement of tumor-infiltrating lymphocytes for predicting likelihood of recurrence in early-stage non–small cell lung cancer. Clin. Cancer Res.

[112] Saltz J (2018). Spatial organization and molecular correlation of tumor-infiltrating lymphocytes using deep learning on pathology images. Cell Rep..

[113] Good Z (2018). Single-cell developmental classification of B cell precursor acute lymphoblastic leukemia at diagnosis reveals predictors of relapse. Nat. Med..

[114] Leelatian, N. et al. High risk glioblastoma cells revealed by machine learning and single cell signaling profiles. Preprint at bioRxiv 632208 10.1101/632208 (2019).

[115] Lou B (2019). An image-based deep learning framework for individualising radiotherapy dose: a retrospective analysis of outcome prediction. Lancet Digital Health.

[116] Nguyen D (2019). 3D radiotherapy dose prediction on head and neck cancer patients with a hierarchically densely connected U-net deep learning architecture. Phys. Med. Biol..

[117] Preuer K (2018). DeepSynergy: predicting anti-cancer drug synergy with deep learning. Bioinformatics.

[118] Koelzer VH, Sirinukunwattana K, Rittscher J, Mertz KD (2019). Precision immunoprofiling by image analysis and artificial intelligence. Virchows Arch..

[119] Geremia E (2011). Spatial decision forests for MS lesion segmentation in multi-channel magnetic resonance images. NeuroImage.

[120] Xu J (2019). A hierarchical integration deep flexible neural forest framework for cancer subtype classification by integrating multi-omics data. BMC Bioinformatics.

[121] Efremova M, Teichmann SA (2020). Computational methods for single-cell omics across modalities. Nat. Methods.

[122] Spitzer MH (2015). An interactive reference framework for modeling a dynamic immune system. Science.

[123] Wang Y (2018). 3D hydrogel breast cancer models for studying the effects of hypoxia on epithelial to mesenchymal transition. Oncotarget.

[124] Saini H (2018). The role of desmoplasia and stromal fibroblasts on anti-cancer drug resistance in a microengineered tumor model. Cel. Mol. Bioeng..

[125] Truong D (2016). Breast cancer cell invasion into a three dimensional tumor-stroma microenvironment. Sci. Rep..

[126] Benam KH (2016). Matched-comparative modeling of normal and diseased human airway responses using a microengineered breathing lung chip. Cell Syst..

[127] Wei Z, Amponsah PK, Al-Shatti M, Nie Z, Bandyopadhyay BC (2012). Engineering of polarized tubular structures in a microfluidic device to study calcium phosphate stone formation. Lab Chip.

[128] Paoli R, Samitier J (2016). Mimicking the kidney: a key role in organ-on-chip development. Micromachines.

[129] Parra ER (2017). Validation of multiplex immunofluorescence panels using multispectral microscopy for immune-profiling of formalin-fixed and paraffin-embedded human tumor tissues. Sci. Rep..

[130] Hoang M (2019). Abstract 753: In situ RNA expression profiling of 1600+ immuno-oncology targets in FFPE tissue using NanoString GeoMxTMDigital Spatial Profiler. Cancer Res..

[131] Codeluppi S (2018). Spatial organization of the somatosensory cortex revealed by osmFISH. Nat. Methods.

[132] Agüi-Gonzalez P, Jähne S, Phan NT (2019). SIMS imaging in neurobiology and cell biology. J. Anal. At. Spectrom..

